# Omega-3 Fatty Acids and Their Interaction with the Gut Microbiome in the Prevention and Amelioration of Type-2 Diabetes

**DOI:** 10.3390/nu14091723

**Published:** 2022-04-21

**Authors:** Manoj Kumar, Namrata Pal, Poonam Sharma, Manoj Kumawat, Devojit Kumar Sarma, Bilkees Nabi, Vinod Verma, Rajnarayan R. Tiwari, Swasti Shubham, Bahram Arjmandi, Ravinder Nagpal

**Affiliations:** 1 National Institute for Research in Environmental Health, Bhopal 462030, India; manoj15ndri@gmail.com (M.K.); namratapal017@gmail.com (N.P.); poonam.mannan91@gmail.com (P.S.); manojkbiochem@gmail.com (M.K.); dkbiotek@gmail.com (D.K.S.); rajtiwari2810@yahoo.co.in (R.R.T.); swasti.shubham@gmail.com (S.S.); 2 Department of Biochemistry and Biochemical Engineering, Sam Higginbottom University of Agriculture, Technology and Sciences, Allahabad 211007, India; bilkeesmir90@gmail.com; 3 Stem Cell Research Centre, Department of Hematology, Sanjay Gandhi Post-Graduate Institute of Medical Sciences, Lucknow 226014, India; vverma29@gmail.com; 4 Department of Nutrition and Integrative Physiology, Florida State University, Tallahassee, FL 32302, USA; barjmandi@fsu.edu

**Keywords:** diabetes mellitus, gut, omega-3 fatty acids, eicosapentaenoic acid, docosahexaenoic acid, alpha-linolenic acid, microbiota

## Abstract

Type-2 diabetes mellitus (T2DM) is often linked with hyperglycemia, disturbed lipid profiles, inflammation, and gut dysbiosis. Omega-3 fatty acid supplementation has a vital role in the management of T2DM. As a result, a better understanding of the potential role of omega-3 fatty acids in the development and progression of T2DM by influencing the intestinal microflora will help to improve the therapeutic intervention for T2DM and related complications. Focusing on the molecular mechanisms and signaling pathways induced by omega-3 fatty acids, this paper attempts to comprehensively review and discuss the putative associations between omega-3 fatty acids, gut dysbiosis, and the pathophysiology of T2DM and its related comorbidities. In addition, we contemplate the importance of gut microbiota in T2DM prevention and treatment and ponder the role of omega-3 fatty acids in T2DM by positively modulating gut microbiota, which may lead to discovery of novel targets and therapeutic strategies thereby paving way for further comprehensive, mechanistic, and clinical studies.

## 1. Introduction

Type-2 diabetes mellitus (T2DM) has become the most pertinent health issue in the current century, affecting approximately 462 million people in 2017 across the world, which correspond to about 6.28% of the global population. Its prevalence is expected to increase by 25% and 51% by 2030 and 2045, respectively. Since T2DM is one of the ten leading causes of death, it is of utmost importance to dig out the preventive as well as treatment measures of the disease and its clinical manifestations/comorbidities [1,2]. It is a severe metabolic disorder marked by insulin resistance resulting in a condition of hyperglycemia accompanied by several comorbidities affecting multiple body organs, including large vessel diseases such as cardiovascular disease (CVD), small vessel diseases including diabetic retinopathy, diabetic neuropathy, non-alcoholic fatty liver disease (NAFLD), and also the conditions affecting cognitive performance and mental health [3,4,5]. The global rise in T2DM could be attributed to inappropriate food consumption and a sedentary lifestyle [6]. It is of paramount importance to have better knowledge and understanding of the role of various dietary components in the pathophysiology of T2DM. Thus, dietary tactics are critical in managing diabetes mellitus [7]. Since 1966, it has been documented that the risk of T2DM has been dramatically lowered with the high fish and seafood intake in northwestern Greenland, possibly due to the action of omega-3 fatty acids (O3-FAs) which comprise a predominant component of fatty acids (FAs) composition of seafood [8,9,10]. Besides, evidence from prospective cohort studies and large prevention programmed trials has demonstrated the protective benefits of anti-inflammatory and antioxidant nutrients such as an O-3 FAs-rich diet [11,12,13,14]. The American Diabetes Association (ADA) recommends a Mediterranean-style diet and long-chain O3-FA without supplementation for diabetic individuals. In the United Kingdom, fish oil consumption without supplements is suggested [15]. Further, the National Lipid Association suggests that the individuals (adults) should consume two or more servings of fish or seafood weekly [16]. According to the Global Burden of Disease Study, the ideal consumption of long-chain O-3 FAs is 0.25 g/d, whereas the global intake averages 0.10 g/d [17].

Leading evidence suggests that the human gut microbiota, which comprises a highly diverse and complex community of thousands of bacterial species, play a vital role in the progression of T2DM. A balanced gut microbiota (homeostasis) plays a fundamental role in a variety of important intestinal functions which, when disturbed, e.g., reduction in intestinal bacterial diversity (dysbiosis), leads to high lipid levels, elevated inflammation, insulin resistance (IR), and thereof high risk of obesity and T2DM [2].

IR, a key factor in the pathology of cardiometabolic diseases, more specifically here T2DM, is also greatly influenced by dietary intake. Disturbances in dietary patterns, e.g., hypercaloric diets, as well as dietary status, contribute to the genesis of IR. On the contrary, hypocaloric diets, diverse feed regimens, and few nutrients have a beneficial effect on IR and disease progression. O-3 FAs, one of the three families of polyunsaturated fatty acids (PUFA), seem to play a protective role against chronic and obesity-associated metabolic diseases, such as IR, T2DM, hepatic steatosis, and CVDs. Eicosapentaenoic acid (EPA, 20:5n−3) and docosahexaenoic acid (DHA, 22:6n−3) are naturally present in fish, while α-linolenic acid (ALA, 18:3n−3) is found in some plant sources such as in leafy greens, rapeseed or canola, nuts, and flaxseed. Considering the significance of the n−6/n−3 PUFA ratio in the inflammatory response, a proper balance in nutritional patterns of n−6/n−3 PUFA is a key determinant in the maintaining of gut microbiota equilibrium [18]. The quality of fatty acids in food also impacts the composition of the gut microbiota, which can affect the host’s metabolic health [18].

Considering the emerging evidence underscoring the role of diet-microbiome interactions in hosts’ health, this narrative review puts together the existing evidence pertaining to the importance of dietary intake of O-3 FAs and its role in the amelioration of T2DM via directly or indirectly modulating the gut microbial community. In addition, we discuss the role of O-3 FAs in the regulation of IR and hence of T2DM while pondering their effect on the gut microbiota (and vice-versa).

## 2. The Role of Omega-3 Fatty Acids in Type-2 Diabetes Mellitus (T2DM)

T2DM is marked by insulin resistance with multifactorial etiology, wherein free fatty acids (FFAs) intake is one of the major factors. FAs’ contribution in the development of IR and T2DM can be better explored by the approach of lipidomic, a less frequently used subcategory of metabolomics [19]. The levels of FFA in the body regulate the insulin signaling pathways (and vice-versa).

**Figure 1:**
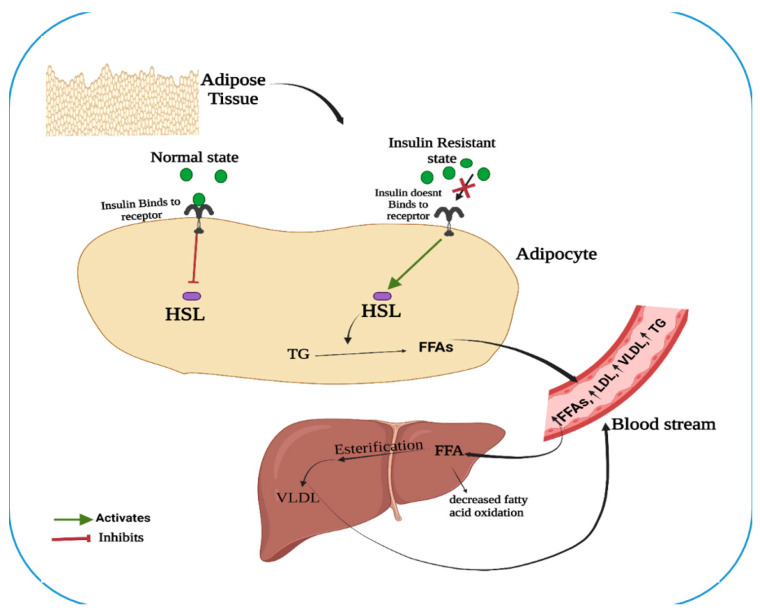
An illustration depicting the relationship between elevated free fatty acids (FFAs) and insulin resistance (IR). (HSL: hormone-sensitive lipase; VLDL: very-low density lipoprotein; LDL: low density lipoprotein; TG: triglycerides).

The interaction of elevated amounts of FFAs and IR might be explained by the binding of insulin to its receptor (Figure 1). Under a normal metabolic state, an enzyme hormone-sensitive lipase (HSL), one of the three lipases present during energy demand in adipose tissue, is tightly controlled by insulin [20] and is inactivated when insulin binds to its receptor. On the contrary, under the state of IR, the insulin fails to bind to its receptor, which in turn activates the HSL and hydrolyses lipids such as triglycerides (TGs), releasing FFA into the circulation to the liver. Hepatocytes take up the FFA and channel them to secretory pathways where another enzyme lipoprotein lipase (LPL) hydrolyses monoglycerides and FFA. This cyclic process goes on to increase the FFA in the blood [19].

The high TGs levels in the body increase FFA levels, which causes the accumulation of DAG and fatty acyl Co- A with elevated reactive oxygen species (ROS). Moreover, this accumulated lipid level is responsible for mitochondrial activity *viz*. β-oxidation of FFA, adenosine triphosphate (ATP) synthesis, and ROS generation. Over time, mitochondria become exhausted, resulting in uncoupling and increased oxidative stress and insulin resistance [21]. Altogether, this activates protein kinase C (PKC) [22] that increases and decreases the phosphorylation of insulin receptor substrate-1 (IRS-1) at serine and tyrosine residues, respectively. This inhibits the activity of Phosphoinositide 3-kinase (PI3K), which disturbs the insulin signaling pathways, eventually resulting in the clinical manifestation of T2DM [19].

Gut microbiota regulates O-3 FA uptake, metabolism and absorption, which is well discussed in the later section. O-3 FA in turn improves T2DM via regulating the insulin signaling in the host (Figure 1). Insulin sensitivity is achieved by the attenuation of ER stress, uncoupling of mitochondria, and improved mitochondrial β-oxidation of fatty acids, thereby cutting down the accumulation of lipid and ROS [23]. Furthermore, they also regulate the secretion of pancreatic β-cell insulin directly by affecting the lipid raft function and structure, and indirectly by restraining and promoting the synthesis of pro-inflammatory mediators (TNF-α, IL-6, IL-17) and adipokines, respectively, in adipose tissue [19]. The exact mechanism of O-3 FAs affecting glucose metabolism is still elusive. However, it has been documented that the defective activity of the key enzymes of O-3 FA desaturation, and D6-, and D5-desaturases, plays an important role in IR occurrence [24].

In T2DM patients, various elements of aging seem to appear earlier or are overrepresented, including consistent inflammation. T2DM patients have higher mortality rate, which is allied to a higher inflammatory score. The cause of the inflammation is undetermined. The senescence-associated secretory phenotype (SASP) has recently been projected as the primary source of inflammation in both aged and T2DM individuals. Senescence and SASP, or oxidative stress and endoplasmic reticulum (ER) stress, have been interrelated to different pathways related to T2DM development and its complications. Recent findings have established a link between oxidative/ER stress and SASP in the context of aging and T2DM, emphasizing endothelial cells dysfunction. Several epidemiological studies stated that a mild inflammation is associated with and may even forecast several age-related diseases (ARDs), including T2DM and its clinical manifestations [25].

O-3-FAs have also shown benefits in several diabetic complications such as CVD, diabetic nephropathy, diabetic neuropathy, and diabetic retinopathy. As reviewed by the American Diabetes Association (January 2016), a diabetic patient will encounter diabetic dyslipidemia, which can be treated with O-3 fatty acids thereby preventing coronary heart disease (CHD). Furthermore, the Australian National Heart Foundation affirmed the role of O-3 FAs in the treatment of hypertriglyceridemia being useful in atrial fibrillation and hypertension, which needs more trials. In the case of diabetic nephropathy, though it has been experimentally proven that O-3 FAs enhance urine albumin-to-creatinine ratio (ACR) and maintain the glomerular filtration rate (GFR). However, more quantified studies are needed for the clinical aspect. Studies have shown promising results in patients with diabetic neuropathy as well as diabetic retinopathy by reducing inflammation and oxidative stress and activation of survival pathways of the cells. Also, retinal angiogenesis has been significantly checked by downregulating the production of several angiogenic factors [26].

Several preclinical studies have demonstrated that O-3 FAs ameliorate various metabolic abnormalities that contribute to the development of diabetes (Table 1). For instance, insulin sensitization is achieved by increased synthesis and release of adipocytokines such as adiponectin and leptin [26,27] and the possibility of preventing IR through anti-inflammatory actions that are mediated directly [28] or by converting to specific pro-resolution mediators such as resolvins and protectins [29,30]. It has also been reported that O-3 FAs may boost fatty acid oxidation and diminish de novo lipogenesis by modulating transcription factors (e.g., sterol regulatory element-binding protein-1c), resulting in reduced hepatic fat storage and maintained hepatic insulin sensitivity [31,32,33,34]. Long-chain O-3 FAs reduce inflammatory pathways by interfering with their enzymatic metabolism. For example, arachidonic acid is converted to pro-inflammatory eicosanoids such as prostaglandins, thromboxane, and leukotrienes. EPA is metabolized to prostaglandins (PGE3), thromboxanes (TXA3), and leukotrienes (LTB5), that act as anti-inflammatory and anti-coagulant molecules [35]. O-3 FAs also possess anti-lipidemic, anti-hypertensive, and anti-coagulant properties, and have recently been shown to modulate the gastrointestinal microbiota [36].

Besides the beneficiary effects of O-3 FAs in context of T2DM, few studies have stated that O-3 FAs interventions do not affect or negatively influence the gut microbiota and trigger the T2DM manifestations (Table 1).

## 3. The Role of Gut Microbiota in the Pathophysiology of Type-2 Diabetes

Humans have various gut microbial enterotypes grouped as “obese” and “lean” that play a major role in the pathophysiology of several disease (e.g., T2DM). Obese microbiota harvests greater energy from the diet resulting in significantly higher increase in total body fat of the host than that of lean microbiota. The gut microbes regulate the host energy equilibrium via conversion of non-fermentable dietary fibers into short-chain fatty acids (SCFAs) facilitating their absorption through intestinal epithelium [56]. Firmicutes, Bacteroidetes, Actinobacteria, Fusobacteria, Verucomicrobia, and Proteobacteria are the dominating taxa in the human gut microbiota, despite differences in composition in different sections of the intestinal tract. The two prominent phyla (Firmicutes and Bacteroidetes) contribute 90% of approximately 1200 bacterial species found in an average adult human gut microbiome. Previous studies have showed that T2DM is associated with gut microbiota dysbiosis at a phylum level, as Firmicutes proportion decreases and Bacteroidetes and Proteobacteria number slightly increases. Previous studies investigated that proportion of butyrate-producing bacteria (e.g., *Roseburia intestinalis* and *Faecalibacterium prausnitzii*) which were lower in T2DM, while *Lactobacillus* spp. and a few opportunistic pathogens, such as *Bacteroides*, *Clostridium* spp., and *E. coli*, were higher in subjects with T2DM. Zhang et al. (2013) conducted a study using 16S rRNA-based high-throughput sequencing and found a decreased abundance of *Akkermansia muciniphila* in subjects with prediabetes or newly diagnosed T2DM, suggesting that a reduced number of this bacteria in the intestine could be used as a biomarker for glucose intolerance [57]. Everard et al. (2013) deciphered the role of *A. muciniphila* that colonizes the intestinal mucous layer and accounts for 3–5% of the human gut microbiota. In obese mice, a daily dosage of viable *A. muciniphila* ameliorated the metabolic endotoxemia, insulin resistance, adipose tissue macrophage infiltration, and glycemia [58].

Generally, prebiotics promotes the growth and/or activity of bacteria in the gastrointestinal (GI) tract. In contrast, the benefits of O-3 FAs on microbiome diversity and composition are still unexplored in human cohorts. DHA supplementation has been proven to improve inflammatory conditions, as well as bacterial dysbiosis in oral and GI diseases. Gut dysbiosis leads to an increase in the level of endotoxins, especially lipopolysaccharide (LPS) in the plasma, a condition called “leaky gut” that results in chronic low-grade inflammation. Elevated plasma endotoxins subsequently activate the inflammasome and augment the inflammatory cytokines’ expression. It has been revealed from the analysis of gut microbial and fecal transfer in mice that the high levels of O-3 FAs increase the production and secretion of alkaline phosphatase in the intestine that induces variations in the gut microbiota composition. The modified gut microbiota reduces the LPS production and gut permeability, thus alleviating the situation of metabolic endotoxemia and inflammation [59].

A randomized, controlled clinical trial [50] showed an improved lipid profile, insulin sensitivity, and decreased atherogenic index following the intake of a probiotic cocktail (VSL#3) along with O-3 FAs. VSL#3 supplementation increased Lactobacilli and Bifidobacteria, and reduced Gram-negative bacteria. The conjugative effect of probiotic + O-3 FAs was greater than probiotic alone in terms of insulin sensitivity and reduced hsCRP. O-3 FAs-rich diet has also been reported to rectify/check the disturbed gut microbiota of drug-naïve T2DM patients, suggesting that there might be a link between microbial composition and O-3 FAs intake [59]. Hutchinson et al. (2020) have suggested that the prebiotic fiber, probiotic bacteria, and O-3 FAs positively modulate this nutrition-inflammation alliance. Such nutritional components interact with gut microbiota and modulate the release of a variety of signaling metabolites [60]. The connections between O-3 FAs and gut microbiota are discussed in the following sections.

## 4. The Intricate Interaction between Omega-3 Fatty Acids and the Gut Microbiome

**Figure 2:**
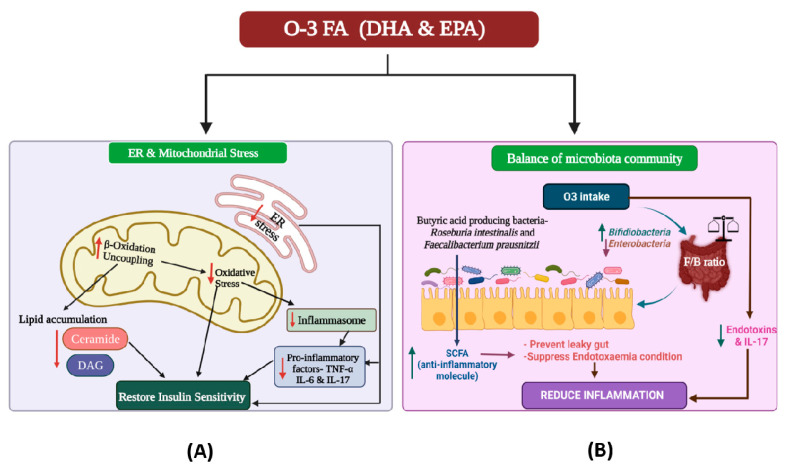
Diagrammatic depiction of the effect of omega-3 fatty acids (O-3 FAs) on (**A**) endoplasmic reticulum (ER) and mitochondrial stress, which results in insulin sensitivity, and (**B**) on gut eubiosis (homeostasis), which results in anti-inflammatory actions [63]. O3: omega-3 fatty acids; EPA: Eicosapentaenoic acid; DHA: docosahexaenoic acid; F/B ratio: Firmicutes-Bacteroidetes ratio; IL: interleukin; DAG: Diacylglycerol; TNF: tumor necrosis factor; SCFA: short-chain fatty acids.

O-3 FAs could affect the gut microbial composition in three different ways: (1) modulating the gut microbial community; (2) altering the pro-inflammatory mediators, viz. endotoxins (lipopolysaccharides) and IL17; and (3) regulating the levels of SCFAs. Dietary intake of O-3 FAs may have a direct effect on the gut microbiota’s diversity and abundance. Studies showed that compared to sunflower oil, fish oil consumption had the highest impact on the diversity of intestinal flora. High O-3 FAs content in fish oil might be responsible for the changes in the gut microbiota pattern due to its inhibitory effect on some of the bacterial strains, which might explicate its health benefits. O-3 FAs are beneficial for gut microbiota as they reduce the growth of *Enterobacteria*, support the growth of *Bifidobacteria*, and subsequently inhibit the inflammation cascade linked with metabolic endotoxemia (Figure 2). Several studies have been conducted on animal models to disentangle the link between fatty acids and gut microbial community. A study conducted on male Sprague Dawley rats showed that the dietary addition of O-3 FAs increases the abundance of gut *Bifidobacteria* [61]. Another study on mice fed with a high-fat diet showed that the EPA and DHA treatment could prevent gut microbiota dysregulation and increase the amount of potentially beneficial lactic acid-producing bacteria and *Bifidobacteria* in the gut. The human gut microbiota is dominated by two major bacterial phyla (Firmicutes and Bacteroidetes) whose (F/B) ratio is significantly increased in subjects with overweight, obesity, NAFLD, and several other diseases. A study on mice fed with a fat-rich diet supported the role of dietary O-3 FAs in positively regulating the F/B ratio (Figure 2) [62]. Menni et al. (2017) investigated the strong association between O-3 FAs and gut microbial composition diversity with specific OTUs and suggested the importance of O-3 FAs supplementation along with prebiotics and probiotics towards a healthy gut [59].

O-3 FAs also play an important role in modulating the gut microbiota by inhibiting the production of pro-inflammatory mediators or helping the synthesis of anti-inflammatory mediators. Lipopolysaccharides (LPS) can penetrate through the intestinal wall under some situations, especially when the barrier is disrupted, causing further damage, due to which the intestinal permeability is altered, and toxic bacterial compounds such as LPS and bacterial DNA accumulate in the hepatic portal circulation. In humans, even modest quantities of LPS in the systemic circulation can trigger an inflammatory response. Gut microbiota is also modulated with varying amounts of SCFAs, thus influencing the gut microbial diversity. SCFAs have been shown to improve systemic inflammation via reducing intestinal permeability and endotoxemia demonstrated to impair insulin signaling and insulin sensitivity. O-3 FAs exert a beneficial effect by restoring the gut microbial community in individuals suffering from several diseases, in addition to increasing the synthesis of SCFAs (anti-inflammatory molecules). By converting non-fermentable dietary fibers into SCFAs such as butyrate, the butyric acid-producing bacteria serve a crucial role in sustaining human gut health. In a study, *Salmonella*-infected mice were given O-3 FAs, which resulted in a significant rise in SCFAs levels, changing the gut microbiota, and favoring host resistance to infections. A prior study also explored that the intake of an O-3 FA-rich diet significantly increases the butyrate-producing microbes including *Blautia*, *Bacteroides*, *Roseburia*, and *Coprococcus* [64].

O-3 FAs strongly influence the intestinal microbes and, likewise, the gut microbiota may directly or indirectly affect the absorption, bioavailability, and biotransformation of O-3 FAs. Gut microbes actively assist in the production of FA-derived metabolites, which may serve as novel active metabolites. Animal model-based studies also supported that the gut microorganisms play a crucial role in the biotransformation of FAs. Bacteria such as *Bacillus proteus* or *Lactobacillus plantarum* transform the O-3 FA precursors α-linolenic acid (ALA) into conjugated linoleic acids (CLA), which later gets hydrogenated to saturated fatty acids such as stearic acid, thereby reducing the composition of PUFA. Also, a wide range of bacteria including lactic acid bacteria (LAB) produce PUFA-derived intermediate metabolites [64,65].

The intestinal flora has a remarkable effect on host health and nutrition-related diseases by regulating the digestion and absorption of PUFAs. The prime source of O-3 FAs is food, and few gut microbes can directly alter the availability of O-3FAs. The gut bacterial genus *Bifidobacterium* regulates fatty acid metabolism or fatty acid uptake by the intestinal epithelium, but the underlying pathway is still undiscovered. Interactively, the intake of O-3 FAs might promote the growth/activities of *Bifidobacterium* in the intestine. In addition, the introduction of probiotics or prebiotics in the diet also raises the relative abundance of *Bifidobacterium* in the gut, which benefits human health by augmenting the level of O-3 PUFA in the blood, providing preventive and therapeutic effects on cardiovascular illnesses and other disorders.

## 5. Role of Gut Microbiota in Alleviating the Inflammatory Responses in T2DM

Although there is a lack of human cohort/clinical studies in this area of research, several preclinical studies on animal models have described the relation of O-3 FAs consumption with changes in the gut microbiota. O-3 FAs, as discussed in the above sections, could directly regulate the gut microbial diversity and abundance. O-3 FA-enriched diet also reduces the colonic abundance of pathogenic *Spirochaetes* and increases that of *Blautia* spp. This eventually inhibits the host inflammatory responses linked with metabolic endotoxemia resulted from ‘leaky gut’. Supplementation of O-3 FAs checks the production of pro-inflammatory cytokines, e.g., IL-17 in monocytes induced by toxic LPS and stimulate the synthesis of anti-inflammatory factors, e.g., IL-10, thereby relieving the inflammation and sustaining the steady state of the gut [65]. As elaborated above, T2D is marked by insulin resistance, which is a consequence of inflammation reactions in the host. Hence, relieving the inflammation may also improve diabetic condition. Conclusively, O-3 FAs modulate T2DM via modifying the diversity and abundance of gut microbiota and inhibiting the inflammatory pathways.

## 6. Combinatorial Strategy to Deal with Type 2 Diabetes

O-3 FAs may regulate the human physiological variables partly by shaping the gut microflora. Several pieces of evidence have implicated the relation between O-3 FAs and gut microbiota. O-3 FAs could affect the intestinal flora and, in turn, gut microbes can also influence the digestion and absorption of O-3 FAs. Supplementation of O-3 FAs has shown substantial changes in the gut microbial community of T2DM patients [65].

## 7. Conclusions and Prospects

T2DM is a serious health problem that presents with a wide array of several other comorbidities. Its prevention, amelioration, and management are sensitively dependent on specific dietary patterns and nutrients. A growing body of evidence suggests that the consumption of O-3 FA may be linked to or even directly confer significant health advantages to patients with T2DM. However, despite the availability of promising empirical evidence over the past decade, the clinical and mechanistic evidence pertaining to the use and effects of omega-3 fatty acids alone or in combination with adjunct strategies (e.g., probiotics, prebiotics, prudent dietary/lifestyle patterns, in specific context to the prevention and amelioration of T2DM and its consequent comorbidities) is currently inconsistent and unclear. Further broader and more inclusive and comprehensive investigations are warranted to determine various factors and elements related to omega-3 fatty acids such as their correct dosage, frequency, personalized application and disparities, and their conjunction with other anti-diabetic approaches including probiotics and prebiotics for prevention, amelioration, and better management of the otherwise ever-growing prevalence and incidence of T2DM among different age groups and populations.

## Figures and Tables

**Table 1:** Tabulated summary of studies demonstrating the effects of Omega-3 fatty acids (from different sources) on gut microbiota, inflammation, and insulin resistance in animals/humans with type-2 diabetes.

Human/Animal Models	Intervention/Treatment	Outcome	References
Fish Oil Capsule (EPA & DHA)			
Offspring of T2DM patients with endothelial dysfunction (n = 50)	Dose: 2 g/d Omega-3 PUFA (EPA + DHA); Fish Oil supplement; Duration: 12 weeks	− Improved endothelial function and reduced proinflammatory markers.	[37]
T2DM patients without prior CVD (n = 97)	Dose: 4 g/d Fish Oil supplement Duration: 12 weeks	− Neutral effect on vascular and metabolic functions. − Improved renal functions.	[38]
Pregnant Women with Type-2 diabetes (n = 88) and healthy women (n = 85)	Dose: 600 mg DHA; Fish Oil supplement Duration: Daily; from early pregnancy till delivery	− Ameliorates red cell membrane anomalies in pregnant women with Type 2 diabetes and neonates	[39]
Patients, who are hypertensive and/or Type2 diabetic obese with high levels of inflammatory markers, (n = 64)	Dose: 1.0 g fish oil supplied in soft gel capsules including 300 mg EPA and 200 mg DHA; Duration: 8 weeks	− Significant reduction in the level of hs-CRP, FBG, and TG after 8 weeks of treatment, whereas no significant changes appeared in IL-6 and TC.	[40]
T2DM patients (BMI ≤ 29.9), aged 25–60 years, with no other chronic diseases, (n = 65)	Dose: 520 mg of DHA + EPA-enriched fish oil each per day; Duration: 24-weeks	− Overall improvement in the lipid profile with a significant decrease in triacylglycerols and atherogenic index − Beneficial effect of EPA + DHA supplementation on waist circumference, glucose, glycosylated hemoglobin, leptin, and leptin/adiponectin ratio	[41]
T2DM patients (n = 40)	Dose: 100 mg/d DHA & 200 mg/d EPA supplement; Durations: 3 months	− Reduction in neuropathic pain symptoms was significantly correlated with an increase in plasma DHA and decrease the level of sphingosine	[42]
Individuals with a high risk of developing diabetes or IFG or IGT (n = 64)	Dose- fish oil capsules (1.2 g DHA + EPA) 2 capsules twice a day; Duration: 12 weeks	− Curcumin and LC n-3 PUFA reduces the insulin resistance (IR) and triglycerides − FA has profound effect on dyslipidemia and Atherogenic index of plasma (AIP)	[43]
T2DM patients with CKD (n = 25)	Dose: 2 g/d concentrated fish oil; Duration: 3 months	− Short term Omega-3 supplementation had no effect on renal function and glycemic control	[44]
Purified O-3 PUFA			
Overweight patients with T2DM (n = 67)	Dose: 2 g purified EPA daily; Duration: 3 months	− Significant decrease in FPG, HbA1c, and HOMA-IR	[45]
T2DM patients with CKD (n = 31)	Dose: Omega-3 PUFA capsules (EPA + DHA) 4 g/d; Duration: 6 weeks	− Non-significant effect on urine albumin excretion; − potential effect of omega-3 supplementation on biomarkers of kidney injury with T2DM	[46]
T2DM nephropathic patients (n = 19)	Dose: OMACOR 3 g/d; Duration: 12 weeks	− No beneficial effect of O-3 FA supplementation on proteinuria; however, it may alter the FA content of erythrocyte membrane FA	[47]
T2DM patients (n = 90)	Dose: 2714 mg/d (EPA = 1548 mg, DHA = 828 mg and 338 mg of other omega = 3 fatty acids); Duration: 2 months	− Significant reduction in HbA1c level	[48]
T2DM with stable coronary artery disease (n = 262)	Dose: 1.86 g/d EPA and 1.5 g/d DHA	− Attenuated progression of albuminuria via conversion of angiotensin enzyme inhibitor or blockage of angiotensin receptor	[49]
O-3 PUFA in combination with probiotics			
Overweight (BMI > 25), healthy adults, aged 40–60 years (n = 60)	Dose: One capsule of VSL#3 and purified omega-3 fatty acid (180 mg EPA and 120 mg DHA per capsule) per daily; Duration: 6 weeks	− Atherogenic index significantly (*p* < 0.01) decreased − Improved HDL, insulin sensitivity, and amelioration of inflammation (hsCRP). − Increase in *Lactobacillus* and *Bifidobacterium* and reduction in gram-negative bacteria	[50]
Patients with NAFLD (n = 48)	Dose: Symbiter Omega—a live multi-strain probiotic mixture with flax and wheat germ oil containing O-3 FA; once daily; Duration: 8 weeks	− Reduced liver fat; improved serum lipids, metabolic profile; and reduced chronic systemic inflammatory state.	[51]
T2DM patients (n = 54)	Dose: Symbiter Omega—a live multi-strain probiotic mixture with flax and wheat germ oil containing O-3 FA; Duration: 8 weeks	− Significant reduction in HOMA2-IR	[52]
O-3 PUFA in combination with Vitamin D			
T2DM patients (n = 1312)	Dose: Vit-D3 2000 IU/d and Omega-3 FA Fish oil supplementation (EPA and DHA) 1 g/d; Duration: 6 h	− Findings do not support the use of Vit-D3 and Omega-3 FA supplementation for preserving kidney function in T2DM patients.	[53]
Pre-diabetic with hypervitaminosis D (n = 168 W)	Dose- 1000 mg omega-3 supplement (360 EPA + 240 mg DHA) twice a day + Vit D 50,000 IU every 2 weeks; Duration: 8 weeks	− Alleviated risk factors of T2DM	[54]
T2DM patients (n = 1312)	Dose: Vitamin D and Omacor (EPA + DHA) 1 g/d; Duration: 5 years	− No effect of Omega-3 FAs on IL-6, hsCRP, or NT-proBNP	[55]

(hs-CRP: high sensitivity C-reactive protein; FBG: fasting blood glucose; T2DM: type-2 diabetes mellitus; TC: total cholesterol; BMI: body mass index; CKD: chronic kidney disease; FPG: fasting plasma glucose; HbA1c: hemoglobin A1C; HOMA-IR: homeostatic model assessment of insulin resistance; NT-proBNP: N-terminal pro b-type natriuretic peptide; O-3 PUFA: omega-3 polyunsaturated fatty acids; EPA: Eicosapentaenoic acid; DHA: docosahexaenoic acid; ALA: α-linolenic acid; NAFLD: non-alcoholic fatty liver disease; FPG: fasting plasma glucose; LC n-3 PUFA: long-chain omega-3 polyunsaturated fatty acids; CKD: chronic kidney disease).

## Data Availability

Not Applicable.

## References

[1] De Silva K., Demmer R.T., Jonsson D., Mousa A., Forbes A., Enticott J. (2022). A data-driven biocomputing pipeline with meta-analysis on high throughput transcriptomics to identify genome-wide miRNA markers associated with type 2 diabetes. Heliyon.

[2] Valder S., Brinkmann C. (2022). Exercise for the Diabetic Gut—Potential Health Effects and Underlying Mechanisms. Nutrients.

[3] McCrimmon R.J., Ryan C.M., Frier B.M. (2012). Diabetes and cognitive dysfunction. Lancet.

[4] Pantalone K.M., Hobbs T.M., Wells B.J., Kong S.X., Kattan M.W., Bouchard J., Yu C., Sakurada B., Milinovich A., Weng W. (2015). Clinical characteristics, complications, comorbidities and treatment patterns among patients with type 2 diabetes mellitus in a large integrated health system. BMJ Open Diabetes Res. Care.

[5] Domingueti C.P., Dusse L.M.S.A., das Graças Carvalho M., de Sousa L.P., Gomes K.B., Fernandes A.P. (2016). Diabetes mellitus: The linkage between oxidative stress, inflammation, hypercoagulability and vascular complications. J. Diabetes Its Complicat..

[6] Bagchi D., Nair S. (2018). Nutritional and Therapeutic Interventions for Diabetes and Metabolic Syndrome.

[7] Hall R.M., Strong A.P., Krebs J.D. (2016). Importance of low carbohydrate diets in diabetes management. Nutr. Diet. Suppl..

[8] Sagild U., Littauer J., Jespersen C.S., Andersen S. (1966). Epidemiological studies in Greenland 1962–1964. I. Diabetes mellitus in Eskimos. Acta Med. Scand..

[9] Bang H.O., Dyerberg J., Sinclair H.M. (1980). The composition of the Eskimo food in north western Greenland. Am. J. Clin. Nutr..

[10] Kromann N., Green A. (1980). Epidemiological studies in the Upernavik district, Greenland. Incidence of some chronic diseases 1950–1974. Acta Med. Scand..

[11] Pitsavos C., Panagiotakos D.B., Tzima N., Chrysohoou C., Economou M., Zampelas A., Stefanadis C. (2005). Adherence to the Mediterranean diet is associated with total antioxidant capacity in healthy adults: The ATTICA study. Am. J. Clin. Nutr..

[12] Schwingshackl L., Missbach B., Konig J., Hoffmann G. (2015). Adherence to a Mediterranean diet and risk of diabetes: A systematic review and meta-analysis. Public Health Nutr..

[13] Martinez-Gonzalez M.A., de la Fuente-Arrillaga C., Nunez-Cordoba J.M., Basterra-Gortari F.J., Beunza J.J., Vazquez Z., Benito S., Tortosa A., Bes-Rastrollo M. (2008). Adherence to Mediterranean diet and risk of developing diabetes: Prospective cohort study. BMJ.

[14] Salas-Salvado J., Bullo M., Babio N., Martinez-Gonzalez M.A., Ibarrola-Jurado N., Basora J., Estruch R., Covas M.I., Corella D., Aros F. (2011). Reduction in the incidence of type 2 diabetes with the Mediterranean diet: Results of the PREDIMED-Reus nutrition intervention randomized trial. Diabetes Care.

[15] American Diabetes Association (2019). 5. Lifestyle Management: Standards of Medical Care in Diabetes-2019. Diabetes Care.

[16] Jacobson T.A., Ito M.K., Maki K.C., Orringer C.E., Bays H.E., Jones P.H., McKenney J.M., Grundy S.M., Gill E.A., Wild R.A. (2015). National lipid association recommendations for patient-centered management of dyslipidemia: Part 1—Full report. J. Clin. Lipidol..

[17] Collaborators G.B.D.D. (2019). Health effects of dietary risks in 195 countries, 1990–2017: A systematic analysis for the Global Burden of Disease Study 2017. Lancet.

[18] Spector A.A. (1999). Essentiality of fatty acids. Lipids.

[19] Shetty S.S., Kumari S. (2021). Fatty acids and their role in type-2 diabetes. Exp. Ther. Med..

[20] Lan Y.-L., Lou J.-C., Lyu W., Zhang B. (2019). Update on the synergistic effect of HSL and insulin in the treatment of metabolic disorders. Ther. Adv. Endocrinol. Metab..

[21] Galiero R., Caturano A., Vetrano E., Cesaro A., Rinaldi L., Salvatore T., Marfella R., Sardu C., Moscarella E., Gragnano F. (2021). Pathophysiological mechanisms and clinical evidence of relationship between Nonalcoholic fatty liver disease (NAFLD) and cardiovascular disease. Rev. Cardiovasc. Med..

[22] Boden G. (2003). Effects of free fatty acids (FFA) on glucose metabolism: Significance for insulin resistance and type 2 diabetes. Exp. Clin. Endocrinol. Diabetes.

[23] Lepretti M., Martucciello S., Burgos Aceves M.A., Putti R., Lionetti L. (2018). Omega-3 Fatty Acids and Insulin Resistance: Focus on the Regulation of Mitochondria and Endoplasmic Reticulum Stress. Nutrients.

[24] Das U.N. (2005). A defect in the activity of Δ6 and Δ5 desaturases may be a factor predisposing to the development of insulin resistance syndrome. Prostaglandins Leukot. Essent. Fat. Acids.

[25] Prattichizzo F., De Nigris V., La Sala L., Procopio A.D., Olivieri F., Ceriello A. (2016). “Inflammaging” as a druggable target: A senescence-associated secretory phenotype—centered view of type 2 diabetes. Oxidative Med. Cell. Longev..

[26] Behl T., Grover M., Shah K., Makkar R., Kaur L., Sharma S., Gupta J. (2019). Role of omega-3-fatty acids in the management of diabetes and associated complications. Bioactive Food as Dietary Interventions for Diabetes.

[27] Perez-Matute P., Perez-Echarri N., Martinez J.A., Marti A., Moreno-Aliaga M.J. (2007). Eicosapentaenoic acid actions on adiposity and insulin resistance in control and high-fat-fed rats: Role of apoptosis, adiponectin and tumour necrosis factor-alpha. Br. J. Nutr..

[28] Talukdar S., Bae E.J., Imamura T., Morinaga H., Fan W., Li P., Lu W.J., Watkins S.M., Olefsky J.M. (2010). GPR120 is an omega-3 fatty acid receptor mediating potent anti-inflammatory and insulin-sensitizing effects. Cell.

[29] González-Périz A., Horrillo R., Ferre N., Gronert K., Dong B., Morán-Salvador E., Titos E., Martínez-Clemente M., López-Parra M., Arroyo V. (2009). Obesity-induced insulin resistance and hepatic steatosis are alleviated by ω-3 fatty acids: A role for resolvins and protectins. FASEB J..

[30] Serhan C.N., Chiang N., Van Dyke T.E. (2008). Resolving inflammation: Dual anti-inflammatory and pro-resolution lipid mediators. Nat. Rev. Immunol..

[31] Tanaka N., Zhang X., Sugiyama E., Kono H., Horiuchi A., Nakajima T., Kanbe H., Tanaka E., Gonzalez F.J., Aoyama T. (2010). Eicosapentaenoic acid improves hepatic steatosis independent of PPARalpha activation through inhibition of SREBP-1 maturation in mice. Biochem. Pharmacol..

[32] Neschen S., Morino K., Dong J., Wang-Fischer Y., Cline G.W., Romanelli A.J., Rossbacher J.C., Moore I.K., Regittnig W., Munoz D.S. (2007). n-3 Fatty acids preserve insulin sensitivity in vivo in a peroxisome proliferator-activated receptor-alpha-dependent manner. Diabetes.

[33] Sato A., Kawano H., Notsu T., Ohta M., Nakakuki M., Mizuguchi K., Itoh M., Suganami T., Ogawa Y. (2010). Antiobesity effect of eicosapentaenoic acid in high-fat/high-sucrose diet-induced obesity: Importance of hepatic lipogenesis. Diabetes.

[34] Jump D.B. (2011). Fatty acid regulation of hepatic lipid metabolism. Curr. Opin. Clin. Nutr. Metab. Care.

[35] De Caterina R., Madonna R., Bertolotto A., Schmidt E.B. (2007). n-3 fatty acids in the treatment of diabetic patients: Biological rationale and clinical data. Diabetes Care.

[36] Watson H., Mitra S., Croden F.C., Taylor M., Wood H.M., Perry S.L., Spencer J.A., Quirke P., Toogood G.J., Lawton C.L. (2018). A randomised trial of the effect of omega-3 polyunsaturated fatty acid supplements on the human intestinal microbiota. Gut.

[37] Rizza S., Tesauro M., Cardillo C., Galli A., Iantorno M., Gigli F., Sbraccia P., Federici M., Quon M.J., Lauro D. (2009). Fish oil supplementation improves endothelial function in normoglycemic offspring of patients with type 2 diabetes. Atherosclerosis.

[38] Wong C.Y., Yiu K.H., Li S.W., Lee S., Tam S., Lau C.P., Tse H.F. (2010). Fish-oil supplement has neutral effects on vascular and metabolic function but improves renal function in patients with Type 2 diabetes mellitus. Diabet. Med..

[39] Min Y., Djahanbakhch O., Hutchinson J., Bhullar A.S., Raveendran M., Hallot A., Eram S., Namugere I., Nateghian S., Ghebremeskel K. (2014). Effect of docosahexaenoic acid-enriched fish oil supplementation in pregnant women with Type 2 diabetes on membrane fatty acids and fetal body composition--double-blinded randomized placebo-controlled trial. Diabet. Med. A J. Br. Diabet. Assoc..

[40] Ellulu M.S., Khaza’ai H., Patimah I., Rahmat A., Abed Y. (2016). Effect of long chain omega-3 polyunsaturated fatty acids on inflammation and metabolic markers in hypertensive and/or diabetic obese adults: A randomized controlled trial. Food Nutr. Res..

[41] Jacobo-Cejudo M.G., Valdes-Ramos R., Guadarrama-Lopez A.L., Pardo-Morales R.V., Martinez-Carrillo B.E., Harbige L.S. (2017). Effect of n-3 Polyunsaturated Fatty Acid Supplementation on Metabolic and Inflammatory Biomarkers in Type 2 Diabetes Mellitus Patients. Nutrients.

[42] Durán A.M., Salto L.M., Câmara J., Basu A., Paquien I., Beeson W.L., Firek A., Cordero-MacIntyre Z., De León M. (2019). Effects of omega-3 polyunsaturated fatty-acid supplementation on neuropathic pain symptoms and sphingosine levels in Mexican-Americans with type 2 diabetes. Diabetes Metab. Syndr. Obes. Targets Ther..

[43] Thota R.N., Acharya S.H., Garg M.L. (2019). Curcumin and/or omega-3 polyunsaturated fatty acids supplementation reduces insulin resistance and blood lipids in individuals with high risk of type 2 diabetes: A randomised controlled trial. Lipids Health Dis..

[44] Usta M., Ersoy A., Ersoy C., Ayar Y., Goksel G., Saka Karagoz İ. (2021). MO235 effect of omega-3 polyunsaturated fatty acid supplementation on glysemic control and renal function in type 2 diabetic patients with chronic kidney disease. Nephrol. Dial. Transplant..

[45] Sarbolouki S., Javanbakht M.H., Derakhshanian H., Hosseinzadeh P., Zareei M., Hashemi S.B., Dorosty A.R., Eshraghian M.R., Djalali M. (2013). Eicosapentaenoic acid improves insulin sensitivity and blood sugar in overweight type 2 diabetes mellitus patients: A double-blind randomised clinical trial. Singap. Med. J..

[46] Miller E.R., Juraschek S.P., Anderson C.A., Guallar E., Henoch-Ryugo K., Charleston J., Turban S., Bennett M.R., Appel L.J. (2013). The effects of n-3 long-chain polyunsaturated fatty acid supplementation on biomarkers of kidney injury in adults with diabetes: Results of the GO-FISH trial. Diabetes Care.

[47] Lee S.M., Chung S.H., Park Y., Park M.K., Son Y.K., Kim S.E., An W.S. (2015). Effect of Omega-3 Fatty Acid on the Fatty Acid Content of the Erythrocyte Membrane and Proteinuria in Patients with Diabetic Nephropathy. Int. J. Endocrinol..

[48] Toorang F., Djazayery A., Djalali M. (2016). Effects of Omega-3 Fatty Acids Supplement on Antioxidant Enzymes Activity in Type 2 Diabetic Patients. Iran. J. Public Health.

[49] Elajami T.K., Alfaddagh A., Lakshminarayan D., Soliman M., Chandnani M., Welty F.K. (2017). Eicosapentaenoic and Docosahexaenoic Acids Attenuate Progression of Albuminuria in Patients With Type 2 Diabetes Mellitus and Coronary Artery Disease. J. Am. Heart Assoc..

[50] Rajkumar H., Mahmood N., Kumar M., Varikuti S.R., Challa H.R., Myakala S.P. (2014). Effect of probiotic (VSL#3) and omega-3 on lipid profile, insulin sensitivity, inflammatory markers, and gut colonization in overweight adults: A randomized, controlled trial. Mediat. Inflamm.

[51] Kobyliak N., Abenavoli L., Falalyeyeva T., Mykhalchyshyn G., Boccuto L., Kyriienko D., Kononenko L., Komisarenko I., Dynnyk O. (2018). Beneficial effects of probiotic combination with omega-3 fatty acids in NAFLD: A randomized clinical study. Minerva Med..

[52] Kobyliak N., Falalyeyeva T., Mykhalchyshyn G., Molochek N., Savchuk O., Kyriienko D., Komisarenko I. (2020). Probiotic and omega-3 polyunsaturated fatty acids supplementation reduces insulin resistance, improves glycemia and obesity parameters in individuals with type 2 diabetes: A randomised controlled trial. Obes. Med..

[53] de Boer I.H., Zelnick L.R., Ruzinski J., Friedenberg G., Duszlak J., Bubes V.Y., Hoofnagle A.N., Thadhani R., Glynn R.J., Buring J.E. (2019). Effect of Vitamin D and Omega-3 Fatty Acid Supplementation on Kidney Function in Patients With Type 2 Diabetes: A Randomized Clinical Trial. JAMA.

[54] Rajabi-Naeeni M., Dolatian M., Qorbani M., Vaezi A.A. (2019). The effect of co supplementation of omega-3 and vitamin D on cardio metabolic risk factors and psychological distress in reproductive-aged women with prediabetes and hypovitaminosis D: A study protocol for a randomized controlled trial. Trials.

[55] Limonte C.P., Zelnick L.R., Ruzinski J., Hoofnagle A.N., Thadhani R., Melamed M.L., Lee I.M., Buring J.E., Sesso H.D., Manson J.E. (2021). Effects of long-term vitamin D and n-3 fatty acid supplementation on inflammatory and cardiac biomarkers in patients with type 2 diabetes: Secondary analyses from a randomised controlled trial. Diabetologia.

[56] Caturano A., Acierno C., Nevola R., Pafundi P.C., Galiero R., Rinaldi L., Salvatore T., Adinolfi L.E., Sasso F.C. (2021). Non-alcoholic fatty liver disease: From pathogenesis to clinical impact. Processes.

[57] Zhang X., Shen D., Fang Z., Jie Z., Qiu X., Zhang C., Chen Y., Ji L. (2013). Human gut microbiota changes reveal the progression of glucose intolerance. PLoS ONE.

[58] Everard A., Cani P.D. (2013). Diabetes, obesity and gut microbiota. Best Pract. Research. Clin. Gastroenterol..

[59] Menni C., Zierer J., Pallister T., Jackson M.A., Long T., Mohney R.P., Steves C.J., Spector T.D., Valdes A.M. (2017). Omega-3 fatty acids correlate with gut microbiome diversity and production of N-carbamylglutamate in middle aged and elderly women. Sci. Rep..

[60] Hutchinson A.N., Tingo L., Brummer R.J. (2020). The Potential Effects of Probiotics and omega-3 Fatty Acids on Chronic Low-Grade Inflammation. Nutrients.

[61] Zhu L., Sha L., Li K., Wang Z., Wang T., Li Y., Liu P., Dong X., Dong Y., Zhang X. (2020). Dietary flaxseed oil rich in omega-3 suppresses severity of type 2 diabetes mellitus via anti-inflammation and modulating gut microbiota in rats. Lipids Health Dis..

[62] Onishi J.C., Campbell S., Moreau M., Patel F., Brooks A.I., Zhou Y.X., Haggblom M.M., Storch J. (2017). Bacterial communities in the small intestine respond differently to those in the caecum and colon in mice fed low- and high-fat diets. Microbiology.

[63] Costantini L., Molinari R., Farinon B., Merendino N. (2017). Impact of Omega-3 Fatty Acids on the Gut Microbiota. Int. J. Mol. Sci..

[64] Shama S., Liu W. (2020). Omega-3 fatty acids and gut microbiota: A reciprocal interaction in nonalcoholic fatty liver disease. Dig. Dis. Sci..

[65] Fu Y., Wang Y., Gao H., Li D., Jiang R., Ge L., Tong C., Xu K. (2021). Associations among Dietary Omega-3 Polyunsaturated Fatty Acids, the Gut Microbiota, and Intestinal Immunity. Mediat. Inflamm..

